# Followership in nurses working in Saudi Arabian hospitals: A cross‐sectional study

**DOI:** 10.1111/nuf.12793

**Published:** 2022-09-02

**Authors:** Sulaiman Alanazi, Richard Wiechula, David Foley

**Affiliations:** ^1^ Adelaide Nursing School, Faculty of Health and Medical Sciences University of Adelaide Adelaide South Australia Australia; ^2^ Nursing Department, Faculty of Applied Medical Sciences Jouf University Jouf Saudi Arabia; ^3^ The Centre for Evidence‐based Practice South Australia (CEPSA): A Joanna Briggs Institute Centre of Excellence Adelaide South Australia Australia

**Keywords:** followership, leadership, nurses, Saudi Arabia, workforce

## Abstract

**Aim:**

To explore the followership styles and their associations with nurses' sociodemographic profiles in Saudi Arabia.

**Background:**

In Saudi Arabia, nurses' role is seen as less important and passive. However, whether they were actually passive followers has not been examined. No previous research has examined nurses' followership styles in Saudi Arabia.

**Methods:**

This cross‐sectional study used a convenience sample of nurses. The Kelley followership questionnaire‐revised was used to determine the prevalence of the five followership styles. Participants' demographic characteristics, which included age, gender, nationality, education level, years of experience, and role, were collected to investigate their associations with followership styles. An online survey was designed and distributed using SurveyMonkey®. Data were analyzed with logistic regression and expressed as odds ratios.

**Results:**

This study included 355 nurses. Findings revealed that the predominant followership style was exemplary (74%), followed by the pragmatist (19%), conformist (4%), and passive styles (3%). Logistic regression analysis revealed that expatriates, higher education, and a leader role had an independent association with an exemplary followership style. Male gender was associated with a passive style. Younger age, male gender, Saudi Arabian nationality, undergraduate qualification, no previous leadership experience, a follower role, and fewer years of experience increased the odds of having a pragmatist style.

**Conclusion and Implications:**

Followership styles were influenced by sociodemographic and work‐related factors. Young nurses with less experience tend to be pragmatist followers. Nursing managers should integrate followership styles when planning leadership and team development courses to ensure maximum team effectiveness as leadership and followership are interdependent.

## 1 INTRODUCTION

Healthcare professionals should be effective in both leadership and follower roles to provide efficient quality care to patients.^1^, ^2^ However, most research on this has focused on leadership roles with limited attention on the followership role.^3^, ^4^, ^5^ Spriggs stated that “while we continue to talk exclusively about leadership, there remain major shortfalls in clinical and health system followership.”^6^,p.637 The field of followership is considered relatively new compared to the well‐established field of leadership. Furthermore, interest is growing in the study of followership among researchers.^7^ Several authors have provided operational definitions to describe followership. Crossman and Crossman defined followership as “a relational role in which followers have the ability to influence leaders and contribute to the improvement and attainment of group and organizational objectives.”^8^,p.484 In healthcare settings, followership, leadership, and communication are considered important nontechnical skills.^9^, ^10^ Hinshaw described followership as “the active abilities of individual members to enhance team performance through task completion, co‐operation and support, constructive challenge where appropriate, and assertiveness.”^10^,p.369 The latter definition is more reflective of Kelley's followership model,^11^ as Kelley places emphasis on the importance of an effective follower's ability to support the leader and the team while simultaneously being able to speak up or challenge them when required.

### 1.1 Kelley's followership model

**Figure 1:**
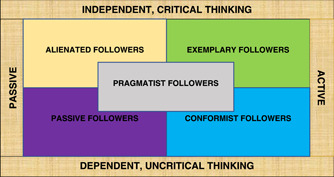
Robert Kelley's followership dimensions and styles (1992) [Color figure can be viewed at wileyonlinelibrary.com]

Kelley's followership model,^11^ Figure 1, on which this study was based, is considered the most important contribution to followership, and has been used in many studies.^12^, ^13^, ^14^, ^15^ Chaleff^16^ and Kellerman,^17^ also important figures in this field, provided similar models. Kelley studied the followership phenomena in individuals working in different organizations for many years. He concluded that an individual's followership style within an organization was determined by two variables: level of engagement (passive or active) and critical thinking (independent or dependent). Kelley created his model, which depicted five followership styles, *exemplary, alienated, pragmatist, conformist, and passive*, each with its own characteristics, as illustrated in Table 1. Kelley maintained that it was the exemplary followers who could make a difference and move organizations toward success.^11^

**Table 1:** Characteristics of the followership styles according to Kelley's model (1992)

Followership styles	Independent thinking	Active engagement	Characteristics & behaviors
Exemplary followers	High	High	Proactive, have strong interpersonal and effective communication skills within the group setting, and play different roles above or below their actual role. Competent, credible, accountable, committed, loyal, motivated, self‐managed, courageous, supportive of the team and the leader, able to speak up or constructively challenge the leader when necessary. Have a deep understanding of the vision, mission, and objectives of the organization.
Alienated followers	High	Low	May hold negative attitudes towards the system or the leader, and this may stem from a feeling that their contributions to the organization are not as recognized or appreciated as they should be. Tend to resist change, and spread negative energy in working groups.
Pragmatist followers	Moderate	Moderate	Fluctuate between the other four followership styles depending on the changing situation and policies of the organization. May lack confidence, and usually put their interests and personal goals first.
Conformist followers	Low	High	“Yes‐people,” they are motivated and supportive of their leaders. However, they depend on the leader to think, instruct, and guide them to achieve the organization's goals. Tend to avoid any conflict with their leaders or team members.
Passive followers	Low	Low	Dependent, lacking critical thinking and less engaged in their work. Need constant monitoring, guidance, and prompts to encourage them to complete their tasks. Tend to avoid challenging or disagreeing with their leaders, even if the decisions or instructions of the leaders are sometimes wrong.

### 1.2 Nursing and followership

Since nurses represent the largest proportion of the healthcare workforce, it is important to understand their followership styles. Abdel Malak stated that “failure to clearly identify the different types of followers and how they impact the leadership is believed to consequently hinder organizational performance in today's work context.”^18^,p.287 In addition, passive or ineffective followership could negatively impact the quality of care and patient safety. The case of Elaine Bromiley showed how ineffective followership can result in severe consequences for patient safety. The young woman died during a minor elective surgery after surgeons lacked situational awareness and lost control. In addition, two nurses felt unable to challenge the surgeons despite knowing the procedure to save the patient's life.^19^, ^20^ Obviously, ineffective followership, including failure to challenge others when they might be wrong, compromises patient safety and contributes to errors occurring in clinical practice.^3^, ^19^, ^20^, ^21^, ^22^ Freeman^23^ asserted that effective followership was an important role for nurses, and called for further research as it was understudied. Therefore, identifying nurses' followership styles could reveal their critical thinking and engagement levels. This could be used by leaders for professional development of their employees.

### 1.3 Nursing in the context of Saudi Arabia

Many leadership scholars have argued that followership concepts, styles, and behavior preferences of leaders may vary across cultures.^7^, ^24^, ^25^, ^26^, ^27^ Saudi Arabia is a country where culture has a major influence on the structure, employment, performance, and relationships between leaders and followers in organizations.^28^ Saudi culture is derived from two major sources: Islamic values and tribal traditions and customs.^28^, ^29^ In this unique cultural context, the nursing profession has confronted many challenges. First, nursing was considered a profession for females, although not suitable for Saudi nationals for religious and cultural reasons, hence the high dependence on expatriates.^29^, ^30^, ^31^ This challenge has largely been overcome. Continuous efforts from the Saudi government, through the Saudization program, have contributed to the acceptance of Saudis to become nurses, although males are still reluctant to join nursing, according to reports from the Ministry of Health.^32^

The second challenge, more relevant to this study, was the cultural‐based view towards the role of nurses in healthcare. Nurses have been seen as less important and less educated compared to other healthcare professionals. Furthermore, their role is perceived to be passive and limited to merely implementing physicians' orders.^29^, ^30^, ^31^ In 2004, a study revealed that Saudi high school students had minimal interest in nursing as a future career compared to medicine and other professions due to cultural and communal values.^33^ Anthony et al. stated that “In 2014 it was still reported that nursing was held in poor regard by Saudis.”^34^,p.3 Another researcher mentioned that “nurses indicated in the interviews that they felt unrecognised as professionals and unappreciated.”^35^,p.196 Thus, nurses were not expected to be leaders or assume active leadership responsibilities. Clearly, the cultural context in Saudi Arabia toward nurses contributed to fostering the perceived power disparities between them and other healthcare professionals, particularly physicians. In a study across 76 countries, which examined the cultural dimensions of power distance in organizations, Saudi Arabia was classified as one of the highest power distant cultures and scored 80% on the Power Distance Index.^27^ In such cultures, independent thinking is discouraged and followers or subordinates are largely dependent on leaders or authority.^26^, ^27^ Schuder also stated that “subordinates in countries with a large power distance are less likely to approach or contradict their superior because people are taught that respect for authority and obedience are priorities.”^26^,p.59

However, health leaders in Saudi Arabia have recognized the challenges that prevent nursing from being perceived as a respected, independent, and legitimate profession. As a collaboration between the Health and Education Ministries, three initiatives were implemented. First, the establishment of educational programs that award bachelor's and master's degrees in nursing in most universities in Saudi Arabia.^34^ Second, additional training courses on leadership and leadership skills have been dedicated to nurses to empower and prepare them to effectively participate in the leadership process at all levels. Third, a scholarship program initiative, where nurses have been sent to developed countries, such as the United States, the United Kingdom, Australia, and Canada,^36^ to obtain higher qualifications and experiences that will enable them to lead or participate in reforming the nursing profession to the highest standards. Therefore, it is valuable for health leaders to understand nurses' followership styles to improve and empower the young nursing workforce in Saudi Arabia.

Based on existing literature, it could be hypothesized that nurses in Saudi Arabia were passive or at best conformist followers according to Kelley's followership model (1992).^11^ To our knowledge, no previous research in Saudi Arabia has described followership in any healthcare profession, including nursing. This is the first study to report on followership of nursing professionals in Saudi Arabia. The study aimed to explore the followership styles of nurses in Saudi Arabia, based on Kelley's followership model,^11^ and investigate the associations between followership styles and participants' sociodemographic profiles.

## 2 METHODS

### 2.1 Study design

This cross‐sectional exploratory study used a convenience sample of nurse professionals. A web‐survey, which comprised the Kelley Followership Questionnaire‐Revised (KFQ‐R),^37^ was used to determine nurses' followership style. Sociodemographic and work‐related data were also collected. The survey was administered via SurveyMonkey® from August to October 2020.

### 2.2 Settings

The study was conducted in public hospitals affiliated with the Ministry of Health (MOH) in Saudi Arabia. Nearly 80% of healthcare services were provided by the MOH.^30^ In addition, MOH hospitals were the major employers for the study's target population. Thus, the sample was representative.

### 2.3 Study participants

Our survey population was nurse professionals from the MOH public hospitals in Saudi Arabia.

### 2.4 Sample size

The total population from which the sample was drawn was (*N*) = 89,093 nurses, according to the annual report from the MOH in Saudi Arabia.^32^ The researcher sent an invitation email, which contained an information sheet regarding the study and a link to the survey, to the General Administration of Nursing at the MOH. The email requested that the invitation be shared with nursing directors in the regions, who would distribute it to the target population locally. Based on the assumption that the invitation reached all nurses, which cannot be verified, the response rate was only 0.6%. This was extremely low and significantly limited the study. The SurveyMonkey® website showed that the survey reached only 508 nurses, with a 70% completion rate. Data collection occurred during the height of the COVID‐19 pandemic, which affected the response rate significantly. Despite the small sample size, the sample composition reflected the population as it included both Saudi and expatriate nurses from two major countries: Philippines and India. Since participants had different native languages, they were expected to communicate in English since it was official language for communication in all Saudi Arabian healthcare organizations.^29^, ^30^, ^31^ Therefore, the study instrument was used in its original language.

### 2.5 Measures

Participants' demographic and work‐related data were categorized as measures to investigate used to association followership styles. These included age (in years), gender (Female, Male), nationality (Saudi, Expatriates), education level (diploma/bachelor, masters/PhD), years of experience in nursing, perceived role (Follower, Leader), and previous leadership position.

### 2.6 Instrument

The Kelley Followership Questionnaire‐Revised (KFQ‐R),^37^ was used to identify the distinct followership styles. The KFQ‐R, a self‐reporting instrument, consisted of 25‐items rated on a 7‐point Likert scale that ranged from 0 (rarely) to 6 (almost always). It measures two subscales, independent critical thinking (13 items) and active engagement (12 items). The KFQ‐R (2019) was a recently revised version of the original Kelley Followership Questionnaire (1992). The revised version was simplified in language, evaluated against social desirability, and validated empirically.^37^ For example, an item in the independent thinking subscale in Kelley's original questionnaire was “Do you help the leader or group see both the upside potential and downside risks of ideas or plans, playing the devil's advocate if need be?” In the revised version, this item was split into “I help the leader to see potential and risks of ideas and plans” and “I help my team to see potential and risks of ideas and plans.”^37^ Another example, an item in the active engagement subscale in Kelley's original questionnaire was “Are you highly committed to and energized by your work and organization, giving them your best ideas and performance?” This was shortened and split in the revised version into “I am committed to my work role” and “I contribute my best at work.” Completing this questionnaire resulted in a participant achieving a plotted score on Kelley's model that indicated one of the five followership styles: exemplary, alienated, pragmatist, conformist, or passive. Internal consistency reliability of the revised tool was evaluated and achieved Cronbach's *α* = .88.^37^ Written permission was obtained from the author to use the revised tool.

### 2.7 Statistical analysis

Descriptive statistics were presented for each outcome as means and standard deviations for normally distributed continuous variables or medians and interquartile ranges for nonnormal continuous variables. Furthermore, frequencies and percentages were used for categorical variables. Mixed‐effects logistic regression was used to model the association between each characteristic of interest and the odds of having each followership style. Logistic regression was used as the dependent variable/s were categorical, such as followership styles. This allowed us to assess the predictive ability of independent variables in predicting or explaining a categorical dependent variable.^38^ The odds ratio (OR) was a measurement that allowed comparing the likelihood of an event (e.g., exemplary followership style) that occurred between two groups (e.g., females and males).^39^ Logistic regression models were fitted with Stata,^40^ and all other analyses were conducted with SPSS.^41^ Results were presented as unadjusted and adjusted ORs and 95% confidence intervals (CIs).

## 3 RESULTS

### 3.1 Characteristics of the study participants

A total of 355 nurses participated, 78% were female (*n* = 278) and the median (interquartile range) age was 33 (9) years. Of these, 48% were expatriate nurses, whereas the MOH recorded only 43% as being expatriate.^32^ Gender proportions were similar to the MOH data, which recorded 76% as females.^32^ Participants' demographic characteristics are described in Table 2 by followership styles.

**Table 2:** Descriptive statistics of the study sample by followership styles

Characteristic	Total (*n* = 355)	Exemplary (*n* = 262)	Pragmatist (*n* = 68)	Conformist (*n* = 15)	Passive (*n* = 10)	MOH data (2020)
Gender, *n* (%)						
Male	77 (22)	43 (56)	23 (30)	4 (5)	7 (9)	21,694 (24)
Female	278 (78)	219 (79)	45 (16)	11 (4)	3 (1)	67,399 (76)
Qualification, *n* (%)						
Masters/PhD degree	75 (21)	64 (85)	9 (12)	2 (3)	0	NA
Diploma/bachelor degree	280 (79)	198 (71)	59 (21)	13 (5)	10 (3)	
Nationality, *n* (%)						
Expatriate	172 (48)	142 (83)	21 (12)	9 (5)	0	38,163 (43)
Saudi national	183 (52)	120 (66)	47 (26)	6 (3)	10 (5)	50,930 (57)
Role, *n* (%)						
Leader	191 (54)	152 (80)	28 (15)	6 (3)	5 (2)	NA
Follower	164 (46)	110 (67)	40 (24)	9 (6)	5 (3)	
Previous leadership position, *n* (%)						
Yes	157 (44)	121 (77)	19 (12)	10 (6)	7 (5)	NA
No	198 (56)	141 (71)	49 (25)	5 (2)	3 (2)	
Age (years) median (IQR)	33 (9 years)					
Experience (years) median (IQR)	10 (10 years)					

Abbreviations: IQR, interquartile range; MOH, Ministry of Health in Saudi Arabia; NA, not available.

### 3.2 Followership styles

**Figure 2:**
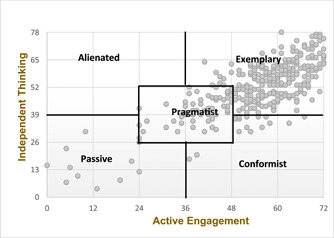
Distribution of the participants' followership styles based on Kelley's model (1992) [Color figure can be viewed at wileyonlinelibrary.com]

Of the 355 participants, 74%, 19%, 4%, and 3% had the exemplary, pragmatist, conformist, and passive followership styles, respectively. The alienated followership style was not found. Figure 2 illustrates the distribution of the participants' followership styles according to Kelley's followership model (1992). Table 3 shows the results of the logistic regression models investigating the association between each characteristic of interest and followership style.

**Table 3:** Logistic regression models of the followership styles

	Univariate		Multivariable		Univariate		Multivariable	
	Exemplary				Conformist			
Characteristic	OR (95% CI)	*p*	OR (95% CI)	*p*	OR (95% CI)	*p*	OR (95% CI)	*p*
Age	1.04 (1.00–1.08)	.051	0.93 (0.86–1.01)	.099	1.01 (0.94–1.08)	.758	1.06 (0.91–1.22)	.467
Gender								
Female	Reference		Reference		Reference		Reference	
Male	0.39 (0.21–0.72)	.003	0.55 (0.25–1.11)	.135	1.33 (0.41–4.30)	0.634	2.12 (0.45–10.02)	.344
Nationality								
Saudi	Reference				Reference		Reference	
Expatriate	2.99 (1.43–6.23)	.004	4.14 (1.64–10.45)	.003	1.63 (0.57–4.68)	.365	2.12 (0.46–9.66)	.332
Previous leadership								
No	Reference		Reference		Reference		Reference	
Yes	1.51 (0.89–2.56)	.128	1.03 (0.56–1.90)	.913	2.65 (0.85–8.19)	.092	4.05 (1.21–13.51)	.053
Qualification								
Diploma/bachelor	Reference		Reference		Reference		Reference	
Masters/PhD	3.61 (1.61–8.10)	.002	4.06 (1.75–9.42)	.001	0.56 (0.12–2.55)	.456	0.63 (0.13–3.11)	.567
Role								
Follower	Reference		Reference		Reference		Reference	
Leader	2.39 (1.39–4.10)	.002	2.49 (1.34–4.64)	.004	0.56 (0.19–1.60)	.279	0.55 (0.17–1.76)	.312
Years of experience	1.06 (1.02–1.11)	.005	1.11 (1.01–1.21)	.031	1.00 (0.92–1.08)	.923	0.91 (0.77–1.09)	.315
	Pragmatist				Passive			
Age	0.95 (0.91–1.00)	.036	1.06 (0.97–1.15)	.228	0.97 (0.86–1.09)	.626	1.01 (0.73–1.38)	.400
Gender								
Female	Reference		Reference		Reference		Reference	
Male	1.97 (1.02–3.81)	.042	1.39 (0.62–3.10)	.420	5.92 (1.28–27.37)	.023	2.26 (0.38–13.47)	.370
Nationality					a			
Saudi	Reference							
Expatriate	0.37 (0.18–0.79)	.010	0.25 (0.10–0.65)	.005		.365		
Previous leadership								
No	Reference		Reference		Reference		Reference	
Yes	0.35 (0.19–0.66)	.001	0.48 (0.24–0.95)	.034	3.02 (0.69–13.14)	.141	4.76 (0.77–29.54)	.094
Qualification					a			
Diploma/bachelor	Reference		Reference					
Masters/PhD	0.33 (0.14–0.79)	.013	0.35 (0.14–0.86)	.022		.456		
Role								
Follower	Reference		Reference		Reference		Reference	
Leader	0.46 (0.26–0.82)	.009	0.50 (0.26–0.96)	.038	0.53 (0.13–2.14)	.374	0.36 (0.07–1.86)	.221
Years of experience	0.93 (0.89–0.98)	.004	0.92 (0.84–1.02)	.107	0.97 (0.86–1.09)	.616	0.96 (0.74–1.24)	.751

Abbreviations: CI, confidence interval; OR, odds ratio.

a No nurses with Passive followership style had a higher degree qualification or were expatriates.

#### 3.2.1 Exemplary followership style

All characteristics except for previous leadership had a statistically significant association with having an exemplary followership style in the univariate analyses. Once all the factors were considered in the multivariable analysis, nationality, qualification, and role all had an independent association. Expatriates had higher odds of having an exemplary style than Saudi Arabian nurses (OR 4.81, 95% CI 2.28–10.14). Nurses with a master's or PhD had higher odds than those with a diploma or bachelor's degree (OR 2.36, 95% CI 1.04–5.33). Leaders had higher odds than followers (OR 2.40, 95% CI 1.31–4.40).

#### 3.2.2 Conformist followership style

There were no statistically significant associations between any characteristics and the conformist followership style.

#### 3.2.3 Passive followership style

Gender had a statistically significant association, with male nurses having higher odds of having a passive style than females (OR 9.17, 95% CI 2.31–36.35). However, due to the large CI this result should be viewed with caution. Models could not be fitted with nationality or qualification since there were no expatriates or people with masters or PhD qualifications with this style.

#### 3.2.4 Pragmatist followership style

Statistically significant associations were found between age, gender, nationality, qualification, previous leadership, role, and years of experience. Increased age resulted in reduced odds of having the pragmatist style (OR 0.96, 95% CI 0.92–1.00). Males had higher odds than females (OR 2.21, 95% CI 1.23–3.95). Expatriates had lower odds than Saudi Arabian nurses (OR 0.40, 95% CI 0.23–0.71). Those with previous leadership experience had lower odds than those without (OR 0.42, 95% CI 0.23–0.75). Nurses with a masters or PhD had lower odds than those with a diploma or bachelor's degree (OR 0.35 95% CI 0.14–0.86). Leaders had lower odds than followers (OR 0.50, 95% CI 0.26–0.96). Increased years of experience resulted in lower odds (OR 0.94, 95% CI 0.89–0.98).

## 4 DISCUSSION

This study aimed to identify the followership styles of nurses in Saudi Arabia using Kelley's followership model (1992) and investigate the association between the followership styles and participants' demographic profiles. Findings revealed that the predominant followership style was exemplary (74%), followed by pragmatist (19%), alienated (0%), conformist (4%), and passive (3%). However, these ratios of nurses' followership styles in Saudi Arabia are quite different from those in other cultures. For instance, in the United States, Boothe et al.,^12^ found 93% of nurses were exemplary, 5% pragmatists, 0% alienated, 2% conformists, and 0% passive. Pack,^42^ also in the United States, reported similar results. However, in Korea and Pakistan, the percentages were different from the United States. A study showed that 18% of Korean nurses were exemplary, 36% pragmatists, 8% alienated, 17% conformists, and 21% passive.^43^ In a sample of resident trainees in Pakistan, 44% were exemplary, 38% pragmatists, 5% alienated, 5% conformists, and 8% passive.^44^ Our percentages were different compared to those in other cultures since our participants were from different cultures: Saudi Arabia, Philippines, and India. If they were from one culture, we may have found different figures. For instance, if we took the Saudi participants separately from expatriates, the figures change to 66% exemplary, 26% pragmatists, 3% conformists, and 5% passive. In addition, we found that the conformist followership style was higher in the Indian participants (7%) compared to the Saudis (3%) and Filipinos (3%), while the passive followership style was found only in Saudi participants. This variance by different nationalities confirmed the statements of Kelley,^45^ Chaleff,^25^ and Carsten et al.,^7^ that followership perceptions and styles differed across cultures. It also validated our logistic regression findings, which revealed that nationality, among other demographic variables, had a statistically significant association with followership styles.

We found that exemplary followership was more likely in expatriate nurses than Saudi nurses. This finding was expected and reflected the reality of nursing in Saudi Arabia, which has depended on expatriate nurses, approximately 80% of the staff, to meet the employment needs.^31^, ^46^ Saudis have become more involved in the nursing profession after the “Saudization” program in 1992, which aimed to reduce the number of foreign workers and replace them with nationals. Saudis currently account for 57% of the total nursing workforce.^32^ Therefore, expatriate nurses with more nursing experience than Saudi nurses are more likely to be exemplary followers, as supported by our multivariable model's statistically significant association of exemplary style with nursing experience.

Older and younger participant age showed a higher odds ratio of being an exemplary or pragmatist follower, respectively. Younger nurses, usually novice or new nurses, are more likely to have less experience. A literature review of novice nurses and their feelings of confusion, uncertainty, stress,^47^ and lack of confidence^48^ found a relationship between young nurses and the pragmatist style, especially when considering the characteristics of Kelley's pragmatist followers.^11^ This indicated that there was a need to increase training and support for young or novice nurses to become exemplary followers more confident in their roles. Young nurses should be educated that a follower role is being active and essential and not being passive or of secondary importance. In addition, simulation training can be an effective method of enhancing followership as stated by Hay‐David et al. “purposeful training of followership in simulation that highlights the key desired attributes is one way to improve such skills in a safe environment.”^49^,p.561

An interesting finding was that gender had a statistically significant association with followership styles. Female and male nurses had higher odds ratios of having the exemplary and passive followership style, respectively. This implied that female nurses were more actively engaged in their roles than male nurses and had higher independent critical thinking scores. This was similar to the findings of the Pakistan study on resident trainees that reported that the frequent predominant followership styles were pragmatist and exemplary in males and females, respectively.^44^ However, these findings were reported as frequencies and not statistically significant figures. The author did not provide an explanation of the differences in followership styles on the basis of gender. In addition, our finding corresponded to a similar finding in a study that investigated the work engagement of nurses in Saudi Arabia and reported that female nurses had higher work engagement scores.^50^ There is no clear explanation for why female nurses are more likely to be exemplary followers than males. This may be explained by nursing being considered as a profession for females.^50^ Male nurses are relatively new, particularly in Saudi Arabia.^31^ The vast majority of male nurses in this sample were Saudi nationals with a lower percentage of exemplary followership style (56%) than female nurses (79%). Percentages of pragmatist, conformist, and passive followership styles were higher in male nurses than female nurses. This should not be ignored by health leaders in Saudi Arabia and an investigation into the factors that impede effective followership, particularly in male nurses, is worth considering.

Education level was found to have a statistically significant association with the exemplary style. Participants with higher education in nursing (master's or PhD degrees) were more likely to be exemplary followers than those with diploma or bachelor degrees. This accorded with Baker et al.,^51^ who investigated followership and leadership characteristics in healthcare professionals in the United States, and found that participants with a higher level of education performed better in the followership performance characteristic of Embracing Change. Education and training are important elements to improve nontechnical skills, such as leadership, followership, and communication. Schwab^52^ found that teaching followership to nursing students enhanced their understanding regarding responsibility, accountability, and power and increased their confidence to engage in exemplary behaviors in clinical practice. Sculli et al.^21^ conducted simulation‐based training, using the Effective Followership Algorithm tool, with nurses and physicians in the United States, who reported higher efficacy and improvement in teamwork and communication skills after the training.

In developed countries, healthcare professionals should be competent in both their clinical and nontechnical skills as most medical errors and adverse events are caused by human factors, such as failure in communication and breakdown in teams.^53^ In a study that addressed challenges in nursing education in Saudi Arabia, the author stated that Saudi graduate nurses had little confidence and lacked communication skills and other essential nursing skills.^46^ Therefore, the quality of nursing education should be improved to accommodate the concepts of nontechnical skills, including effective leadership, followership, and communication, especially in the diploma and bachelor programs.

The perceived roles of follower or leader also had a statistically significant association with two followership styles. In this study, participants were asked to answer the question: “In your current role for the majority of your practice, would you consider yourself as follower or leader?” In response, 54% and 46% perceived their role as leaders, even though most were not in a leadership position, and followers, respectively. Those who perceived their role as leaders and followers had higher odds of having an exemplary and pragmatist style, respectively. Participants who perceived themselves as leaders were more confident, actively engaged in their role, and had the potential to assume leadership responsibilities. This was consistent with Kelley's^11^ and Chaleff's^16^ descriptions of exemplary or effective followers as willing to be leaders or followers and have the courage to assume responsibility. Kelley,^11^ Baker et al.,^51^ and many leadership scholars assert that the characteristics or attributes of effective or exemplary leaders are the same characteristics of exemplary followers. Pragmatist followers are reluctant to describe themselves as leaders or unwilling to assume leadership responsibilities. Interestingly, our results indicated that not having a previous leadership position had a statistically significant association with the pragmatist followership style. These findings were consistent with Kelley's and validated the theory that pragmatist followers who had moderate levels of active engagement and independent critical thinking lacked confidence and subsequently required support and encouragement to reach their potential. Education and simulation training can be useful tools to promote exemplary or effective followership behaviors.^21^, ^49^, ^52^

This study raised some questions to be answered to better understand followership more deeply in the context of nursing in Saudi Arabia. For instance, is there a difference in the perception of followership between female and male nurses and between expatriates and Saudi nationals? What characteristics and behaviors are present in both followers and leaders? Do nursing leaders prefer and encourage the exemplary followership style in their followers, and if they do, why? What challenges exist that prevent or reduce the practice of exemplary or effective followership? These questions are best answered using qualitative approaches, planned as a second phase of this study.

### 4.1 Limitations

An important limitation of this study was that followership style might determine completion of the survey as this was a convenience or self‐selected sample. Consequently, nurses with particular followership styles may be more likely to participate, which could have resulted in responder bias. Given that the sample was small and not randomized, the findings lacked reliability and validity. Only generalizations from the information can be made. Therefore, this study should be replicated with a larger sample size and additional efforts should be made to reduce and control for responder bias.

## 5 CONCLUSION AND IMPLICATIONS

This was the first study to report on followership styles in nursing professionals in Saudi Arabia. This study revealed that followership styles were influenced by sociodemographic and work‐related factors. For instance, gender, nationality, education, and experience had significant associations with followership styles.

These findings might assist nurse managers to identify nurses with characteristics likely to be associated with exemplary followership style and allocate them in the desired departments, particularly critical ones, such as emergency or operating rooms. Simultaneously, the findings might assist in identifying nurses with characteristics more likely to be associated with ineffective followership styles to be mentored and trained. Nursing leadership should consider conducting followership assessment before recruiting nurses in critical departments. This is important since, for example, a nurse with a passive or conformist followership style is highly likely not to challenge erroneous decisions made by others, which could result in tragic consequences, such as Elaine Bromley's case. In addition, nursing managers should consider integrating followership when planning for leadership and team development to ensure maximum team effectiveness as both leadership and followership are interdependent. Similarly, for undergraduate nursing education, introducing leadership and followership will enable nursing students to build these important nontechnical skills at an early stage.

Further research on followership in the nursing profession of Saudi Arabia is required to fully understand this concept and its relationship with other important variables, such as patient outcomes, quality of nursing services, or organizational effectiveness. Finally, the Kelley Followership questionnaire‐revised tool,^37^ was effective in identifying the followership styles according to Kelley's followership model. Thus, we recommend using this tool in the study of followership styles as it is empirically validated, evaluated against social desirability, and has simplified language and points of criticism in Kelley's original followership questionnaire.

## CONFLICT OF INTEREST

The authors declare no conflict of interest.

## ETHICS STATEMENT

Informed consent for participation was obtained within the survey from all the participants. This study was approved by The University of Adelaide Human Research Ethics Committee (approval number: H‐2020‐026) and the Central Institutional Review Board at the Ministry of Health in Saudi Arabia (log number: 20‐161E).

## Data Availability

The data that support the findings of this study are available from the corresponding author upon reasonable request.
